# Layered Double Hydroxide Nanocomposite Coatings for Improved Flame Retardancy of Polyethylene-Based Copolymers

**DOI:** 10.3390/polym17233189

**Published:** 2025-11-29

**Authors:** Giuseppe Trapani, Rossella Arrigo, Michele Sisani, Maria Bastianini, Alberto Frache

**Affiliations:** 1 Department of Applied Science and Technology, Politecnico di Torino, Viale Teresa Michel 5, 15121 Alessandria, Italy; giuseppe.trapani@polito.it (G.T.); rossella.arrigo@polito.it (R.A.); 2 Prolabin&Tefarm, Ponte Felicino, 06134 Perugia, Italy; michele.sisani@prolabintefarm.com (M.S.); maria.bastianini@prolabintefarm.com (M.B.)

**Keywords:** ethylene–vinyl acetate, ethylene–butyl acrylate, cast extrusion, cone calorimeter, combustion behavior

## Abstract

This work proposes a coating approach for obtaining flame-retardant ethylene–vinyl acetate (EVA) and ethylene–butyl acrylate (EBA) copolymer-based materials. Nanocomposite films of EVA and EBA were first produced by cast extrusion, with two types of layered double hydroxides (LDHs) differing in the aspect ratio used as nanofillers. Subsequently, the films were applied as a coating to the corresponding neat copolymer substrate, and the combustion behavior of the so-obtained samples was evaluated through cone calorimeter tests. Despite the small amount of nanofillers (0.5 wt.% considering the whole specimen), the application of the coatings significantly improved the time to ignition compared to the pristine copolymers, while the shape of the heat release rate curves and the relative peak values remained relatively unchanged. The effect of the embedded nanofillers in delaying the ignition was more effective for the EVA-based systems than for the EBA ones (showing an increment of 30% and 12%, respectively, compared to the uncoated samples), likely due to the more homogeneous dispersion of the LDHs obtained in the first case. The obtained results demonstrate the effectiveness of the coating approach, since it allows the flame-retardant action to be concentrated on the surface of a polymer system, where combustion specifically takes place, while minimizing the required amount of flame retardant.

## 1. Introduction

Thermoplastic polymers are renowned for several key characteristics, such as their easy processability, low cost compared to other materials, lightweight, and versatility. However, one of the most severe issues of these materials is undoubtedly their high flammability [1] and all the consequent concerns in terms of risk to the health and life of people involved in fires and the pollution produced by the resulting fumes. Various approaches have been developed to overcome these issues, from the chemical functionalization of polymer chains [2] to additives and fillers being introduced through a compounding step. The most commonly employed flame-retardant additives include metal hydroxides [3], halogenates [4,5], phosphorus compounds [6], intumescent systems [7,8], and nanoparticles [9,10], with the possibility of exploiting the synergy between different systems [11,12].

Ethylene–vinyl acetate (EVA) and ethylene–butyl acrylate (EBA) copolymers are commodities that are widely utilized in the fabrication of sheaths for electrical cables, pipes, and films. These materials are therefore employed in a range of applications where the flame-retardant properties of the materials are required [13]. Concerning EVA, there is a large number of studies in the literature dealing with the development of several strategies for improving its flame retardancy through the introduction of additives and/or fillers in the bulk [14,15,16,17,18]. For instance, Camino et al. [19] compared the behavior of EVA-based systems containing a layered double hydroxide (LDH), magnesium hydroxide, aluminum hydroxide, or boehmite, demonstrating that the utilization of hydrotalcites caused a significant decrease in the time to ignition compared to the pristine matrix. Furthermore, it was shown that, similarly to conventional magnesium and aluminum hydroxides [20,21], the water molecules trapped in between the LDH layers can dilute the flammable gases during pyrolysis, thus inhibiting flame propagation in burning materials. Additionally, in the same work, it is reported that LDHs are also able to form a mixed oxide residue that leads to an insulating char layer acting as additional protection for the underlying polymer. In contrast, for EBA, there are only a few works discussing approaches for flame retardancy [22,23]. This copolymer, however, can be considered a superior alternative to EVA, given its reduced polarity, attributable to the length of the butyl groups compared to the acetate ones of EVA. This lower polarity results in reduced hygroscopic tendency [24], a property that can be pivotal in specific applications, such as cable sheathing.

Regardless of the specific polymer, the most prevalent approach to flame retardancy entails the incorporation of high concentrations (typically a minimum of 50 wt%) of flame-retardant additives. This strategy, however, gives rise to two direct consequences. Firstly, it is evident that the mechanical properties of the material are subject to a complete alteration. In the case of EVA and EBA, there is an evident transition from a flexible to a significantly more rigid and brittle behavior [19], which is not generally desirable, particularly in applications such as wire coatings, where flexibility is an essential attribute that must be preserved. On the other hand, the incorporation of high loadings of inorganic fillers has been demonstrated to result in a significant alteration in the average density of the material. This, in turn, has a significant impact on the overall weight of the final object [25]. In order to circumvent these issues, an alternative approach has been proposed, namely the localization of flame retardants on the surface of the polymer, where combustion specifically takes place. In this context, Colonna et al. [26] demonstrated that the time to ignition of an EVA-based system containing organomodified nanoclays can be significantly enhanced through an aging treatment at a temperature close to the melting point, which promotes an easier migration of the clay toward the surface during the early stage of combustion. The strategy of exploiting protective coatings for flame retardancy has been extensively reviewed by Malucelli et al. [27], showing that the presence of a coating on the polymer surface, capable of regulating the heat and mass flows, allows the fire behavior of the material to be governed. Additionally, Matta et al. [28] showed that the localization of the flame retardant on the polymer surface promotes an initial shielding effect that causes a postponement in the time to ignition and the time to peak.

In this work, nanostructured films based on EVA and EBA and containing two types of LDHs characterized by different dimensions were formulated through cast extrusion and applied as coatings on unfilled substrates. The aim was to assess their influence on the combustion behavior of the two copolymers. After a preliminary characterization of the films’ microstructure, coated specimens were produced and subjected to cone calorimeter tests. The obtained results highlighted an increase in the time to ignition compared to the uncoated samples, especially for the EVA-based systems, confirming the effectiveness of the proposed method to improve fire safety.

## 2. Materials and Methods

### 2.1. Materials

The polymers used in this work as matrices were (i) ethylene–butyl acrylate (EBA) Lucofin^®^ 1400MN (supplied by Lucobit, Wesseling, Germany), with a butyl acrylate content of 17%, and (ii) ethylene–vinyl acetate (EVA) Greenflex MQ 40 BA (by Versalis, San Donato Milanese, Italy), with a vinyl acetate content of 19%. Both copolymers are characterized by a melt flow index of 7.0 g/10 min (190 °C, 2.16 kg).

Two types of layered double hydroxides (LDHs) with different dimensions and modified with oleate groups ([Mg_0.66_Al_0.34_(OH)_2_](C_18_H_35_O_2_)_0.34_ × 0.9 H_2_O) were used, both synthesized by Prolabin&Tefarm (Perugia, Italy). Hereinafter, the LDHs are referred to as S-LDHs (D_50_ = 5.60 μm, D_90_ = 28.78 μm) and L-LDHs (D_50_ = 18.67 µm, D_90_ = 48.91 µm).

### 2.2. Processing and Sample Preparation

The EBA- and EVA-based nanocomposite films were produced through melt compounding and cast extrusion using a twin-screw extruder Thermo Fisher Scientific™ Process 11 (Waltham, MA, USA) equipped with a rectangular-shaped die (thickness = 1 mm, width = 25 mm) and a Process 11 Sheet Take Off Unit (Thermo Fisher Scientific™ Waltham, MA, USA). The selected processing conditions were as follows: screw rotation speed of 150 rpm, feed rate of 500 g/h, and temperature profile of 160-165-170-180-190-190-190 °C and 140-160-170-180-180-180-180 °C for EBA- and EVA-based materials, respectively.

The so-produced nanocomposite films (LDH content: 5 wt%) were characterized by a thickness of about 280 µm.

A Collins (Maitenbeth, Germany) P200T hot plate press, operating at 190 °C and 100 bar, was used to produce 50 × 50 × 3 pristine copolymer specimens, which were used as substrates to produce coated samples and as a reference for all the characterizations. To this aim, the films obtained via cast extrusion were applied to the compression-molded specimens of pristine EBA and EVA through a further compression molding process using a Collins (Maitenbeth, Germany) P200T hot plate press at 85 °C and 20 bar for 30 s.

### 2.3. Characterization Techniques

Thermogravimetric analyses were performed on samples of 10 ± 1 mg using a Discovery TGA 5500 (TA Instrument, New Castle, DE, USA); the samples were heated from 50 to 700 °C at 10 °C/min under air flow (25 mL/min). T_onset_ (temperature at which 2% of weight loss occurs) and T_max_ (temperature at which maximum weight loss rate is observed in dTG (derivative) curves) were evaluated.

The thermal characterization of the coating films was performed using a DSC TA Q20 (TA Instruments, New Castle, DE, USA) with the following thermal cycle (performed under a nitrogen atmosphere): a first heating scan from −70 °C to 200 °C, an isothermal step at 200 °C for 3 min, a cooling scan from 200 °C to −70 °C, and a second heating ramp from −70 °C to 200 °C. During all the steps, the rate of heating/cooling was maintained at 10 °C/min. The melting temperature (T_m_) and the melting enthalpy (∆H_m_) of all the samples were evaluated in the first heating scan. Additionally, the crystallinity content ($\chi_{c}$) was evaluated, taking into account that EBA and EVA are polyethylene-based copolymers in which only the polyethylene fraction is able to crystallize, while butyl acrylate and vinyl acetate fractions are amorphous. $\chi_{c}$ was calculated using the following formula:

(1) $$\chi_{c}=\frac{\Delta H_{m}}{PE\%\cdot \Delta H_{m}^{0}\cdot \;\left(1-x\right)}\cdot 100$$

where $\Delta H_{m}$ is the melting enthalpy, PE% is the polyethylene content of EBA and EVA (0.83 and 0.81, respectively), $\Delta H_{m}^{0}$ is the melting enthalpy of the 100% crystalline polyethylene (277 J/g [28]), and x represents the nanofiller content.

The rheological behavior of the unfilled and nanocomposite films was evaluated in an inert atmosphere (N_2_) using an ARES (TA Instruments, New Castle, DE, USA) strain-controlled plate–plate rheometer. Frequency sweep measurements were performed at 190 or 180 °C for EBA- or EVA-based materials, respectively. For the unfilled films, the tests were carried out from 100 to 0.1 rad/s, while a frequency range of 100–0.01 rad/s was selected for the nanocomposites. The strain amplitude was selected for each sample to fall in the linear viscoelastic region (preliminary assessed through strain sweep tests). The complex viscosity data as a function of the frequency were fitted using the following Cross-like model modified to take into account the yield stress [29]:

(2) $$\eta^{*}\left(\omega \right)=\frac{\eta_{0}}{\left[1+{\left(\lambda \omega \right)}^{1-n}\right]}+\frac{\sigma_{0}}{\omega }$$

where $\eta^{*}$ is the complex viscosity, ω is the frequency, $\eta_{0}$ is the zero-shear viscosity, λ is the relaxation time, *n* is the flow index, and $\sigma_{0}$ is the yield stress.

A Zeiss (Oberkochen, Germany) EVO 15 Scanning Electron Microscope (SEM) (20 kV beam voltage and 8.5 mm working distance) was used for morphological analysis. The sections to be observed were obtained by fracturing the films (in the direction parallel to the machine direction) in liquid nitrogen. The samples were gold sputtered prior to morphological analysis.

The combustion behavior was evaluated with a Noselab ATS (Monza, Italy) cone calorimeter, according to the ISO5660 standard. EBA-based specimens were tested under a radiant heat flux of 25 and 30 kW/m^2^, while for EVA-based materials, a heat flux of 30 kW/m^2^ was used. To ensure reliability, three replicates were performed for each system, allowing for the estimation of the time to ignition (TTI), the heat release rate (HRR), the total heat release (THR), the peak of the heat release rate (pkHRR), and the time of the pkHRR (tpeak). In addition, to evaluate the fire hazard, the fire growth rate index (FIGRA) was calculated according to Equation (3) [30], while for the fire performance index (FPI), Equation (4) was used [31]:

(3) $$FIGRA=\frac{pkHRR}{t_{peak}}$$

(4) $$FPI=\frac{TTI}{pkHRR}$$

## 3. Results

### 3.1. Preliminary Characterization of EVA- and EBA-Based Nanocomposite Films

**Figure 1:**
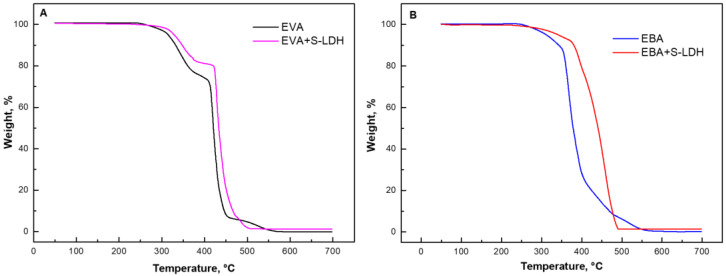
TGA curves for frequency for (**A**) EVA- and (**B**) EBA-based materials containing S-LDHs.

**Figure 2:**
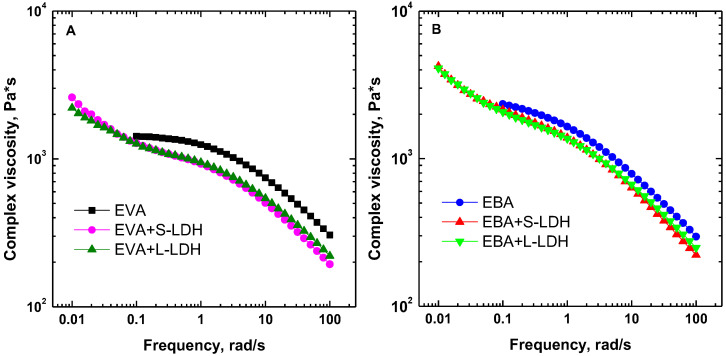
Complex viscosity curves as a function of the frequency for (**A**) EVA- and (**B**) EBA-based materials.

The thermo-oxidative degradation of EVA- and EBA-based nanocomposites was evaluated through TGA analyses in an oxidative atmosphere; the obtained curves are depicted in Figure 1, while the main results are listed in Table 1. Unfilled EVA (Figure 1A) undergoes three degradation steps. According to the literature [16,32], the first step, occurring between 290 and 390 °C, is associated with the evolution of acetic acid. The second stage (occurring between 400 and 470 °C) is attributed to the decomposition of the ethylene-co-acetylene random copolymer resulting from deacylation. Finally, for 490 to 580 °C, the oxidation of the products formed during the previous steps occurs. Also, for EBA (Figure 2B), three main degradation stages can be identified [22,33]. In this case, the first step (250–340 °C) is associated with the cleavage of the butyl acrylate chains, the second one (340–430 °C) is attributed to the chain scission of the polymer backbone and further oxidation of the previously formed unsaturated fragments, and the last decomposition step involves the oxidation of the remaining residue.

The incorporation of both types of LDHs (the results for the films containing S-LDH are reported in Figure 1A,B and Table 1) results in an enhancement of the thermo-oxidative resistance of both copolymers. Specifically, the EVA-based nanocomposite exhibits an increase in both T_onset_ and T_max_ compared to the unfilled matrix. The effect of the nanofillers is even more considerable for EBA+S-LDHs, as highlighted by an increase of approximately 14 °C in T_onset_ and of 55 °C in T_max_ with respect to the neat copolymer. As widely reported in the literature, the observed delay in the thermo-oxidative phenomena of the nanocomposites can be attributed to the barrier to oxygen and to the volatile degradation products generated by the embedded nanofillers, as well as to their thermal insulating action [34,35,36].

Table 2 presents the main results of the thermal characterization of the nanocomposite films. The thermograms recorded during the first heating scan are reported in Figures S1 and S2. According to the literature [37], both pristine copolymers show two melting peaks, corresponding to the primary and secondary crystallization. From a general point of view, the introduction of the nanofillers does not significantly affect the melting temperatures of the unfilled matrices or the crystallinity content.

The complex viscosity curves as a function of the frequency obtained through frequency sweep tests are reported in Figure 2A,B. Both copolymers show the expected pseudo-plastic trend, involving the appearance of a Newtonian plateau in the low-frequency region, followed by a shear-thinning behavior at high frequencies. It should be noted that EBA, especially at low frequencies, shows higher viscosity values than EVA and less pronounced Newtonian characteristics, likely due to the different molecular weights of the two copolymers. The incorporation of the nanofillers causes the disappearance of the Newtonian plateau in the low-frequency region; in fact, all the investigated nanocomposite films exhibit a yield stress behavior, associated with the establishment of copolymer–filler and filler–filler interactions that hinder the complete relaxation of the copolymer macromolecules [38].

Looking at the high-frequency region, lower complex viscosity values are obtained for the nanocomposite films compared to the unfilled copolymers. According to the literature [39], this behavior can be explained by considering a preferential orientation of the nanofillers along the shear flow direction. Lastly, it should be noted that the different aspect ratios of the LDHs do not affect the rheological response of the nanocomposites. In fact, for both EVA- and EBA-based systems, practically overlapping viscosity curves are obtained as a result of the incorporation of S-LDHs or L-LDHs.

**Figure 3:**
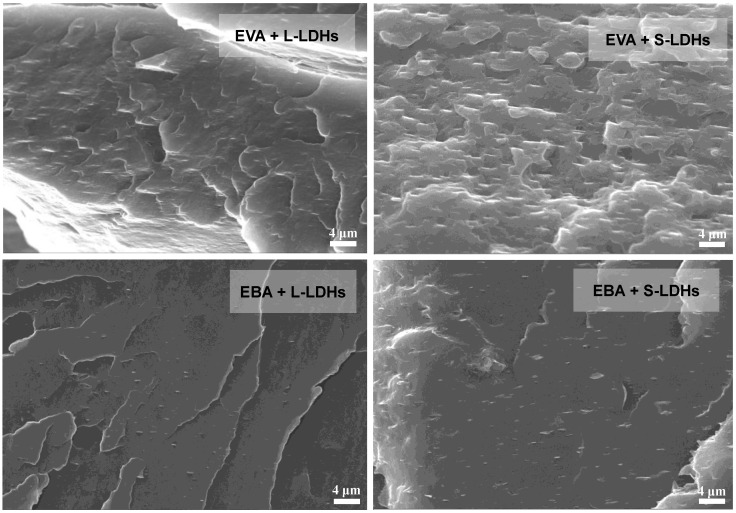
SEM micrographs for EVA- and EBA-based nanocomposite films.

From the SEM micrographs shown in Figure 3, it is evident that, regardless of matrix and type of filler, the lamellar nanofillers appear, in general, well dispersed and well distributed. In fact, in both matrices, the nanofillers are mainly dispersed as thin platelets with a thickness of less than a micrometer. Furthermore, a clear, strong orientation of the nanofillers in the direction of the production of the films (i.e., machine direction) can be observed, mainly attributable to the application of the non-isothermal elongation flow during the cast extrusion and calendering processes.

**Figure 4:**
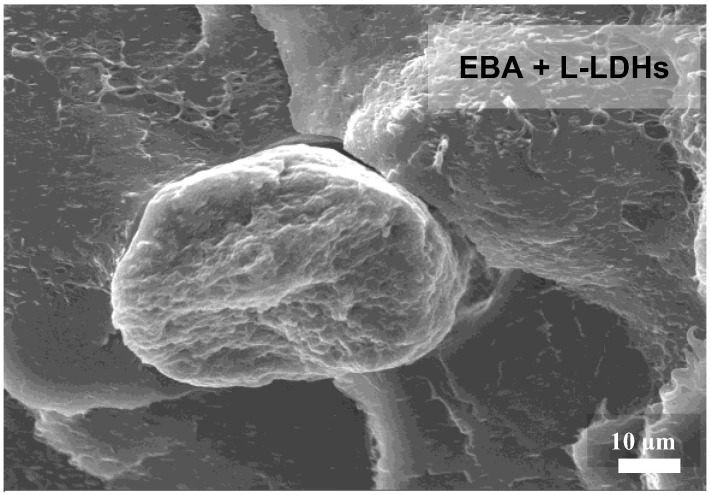
SEM micrographs of EBA-based nanocomposite film containing L-LDHs showing a nanofiller aggregate.

However, although rheological analyses suggested an excellent extent of dispersion/distribution of the nanofillers in both matrices, it should be noted that EBA-based nanocomposites exhibit the presence of some large-sized aggregates, as illustrated in the micrograph presented in Figure 4. Thus, it seems that the LDHs dispersion in EBA is less effective than in EVA, likely because of the different polarity of the copolymers. In particular, owing to the presence of butyl groups, EBA is less polar compared to EVA, making this copolymer less compatible with the embedded nanofillers.

### 3.2. Combustion Behavior of Coated Samples

**Figure 5:**
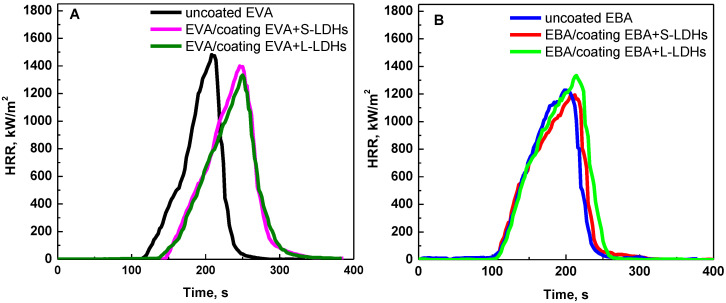
HRR as a function of time: cone calorimetry (heat flux = 30 kW/m^2^) curves of (**A**) EVA- and (**B**) EBA-coated samples. The curves for uncoated EVA and EBA specimens are also reported.

As already illustrated in the experimental section, the EVA- and EBA-based films were applied to compression-molded specimens of pristine copolymers through a further compression molding process, and the combustion behavior of the so-obtained coated samples was evaluated through cone calorimeter tests with an incident heat flux of 30 kW/m^2^. The obtained curves of HRR as a function of time for uncoated and surface-coated samples are reported in Figure 5A,B for EVA- and EBA-based materials, respectively. Additionally, THR curves are depicted in Figures S3 and S4, and Table 3 reports the most relevant data and parameters collected during these tests.

Concerning EVA-based samples, it is evident that the application of surface coatings results in a significant delay in the ignition of the specimens. This phenomenon can be noticed by looking at the HRR curves of the coated specimens, which are shifted forward by about 30 s, irrespective of the type of LDHs. Furthermore, the shape of the curves remains almost unchanged compared to that of the uncoated EVA sample. In fact, once the protective action of the surface coating is exhausted, the underlying pure EVA specimen undergoes almost complete combustion, resulting in a negligible final residue.

The increase in TTI for samples coated with a surface film containing nanoclays has already been reported in the literature [9,40,41]. In particular, it has been demonstrated that during the heating of the material, the ablation of polymer chains promotes the migration of the lamellae towards the surface to form a physical ceramic barrier that limits the heat and oxygen fluxes towards the underlying polymer. Concurrently, the coating also acts as a barrier for the volatile gases resulting from the thermal degradation of the underlying polymer. It has been established that, due to the low thickness of the barrier layer and the minimal filler content, its protective efficacy is restricted to the early stage of the cone calorimeter test. In fact, due to the increasing pressure of volatiles produced by the substrate, the rupture of the film occurs, and the process proceeds through the ignition of the gas phase and the subsequent combustion of the polymer, with the same mechanism as uncoated materials. However, it should be noticed that the surface localization of the nanofillers on the polymer surface has a beneficial effect on TTI with respect to the classical bulk incorporation. In fact, in this last case, an anticipation of ignition is often observed compared to unfilled polymers [42].

In contrast, for EBA-coated samples, a less relevant delay in TTI is observed. This result can be explained by considering the poorer dispersion of the LDH nanoparticles in this copolymer compared to EVA, as assessed through morphological analyses. In particular, the presence of LDH aggregates in EBA results in a reduced number of mobile lamellae available to form the physical barrier in close proximity to the specimen surface [41]. Furthermore, the different TTI of the two uncoated copolymers should also be considered; in particular, the results of the cone calorimeter tests demonstrate that the ignition is anticipated for EBA compared to EVA. Therefore, the different effectiveness of the surface coating in the two cases can also be explained by the fact that, under a heat flux of 30 kW/m^2^, the rate at which the LDHs lamellae arrange to form the protective layer is lower than the EBA ignition. In order to verify a possible protective effect of the surface coating also for EBA-based systems, the cone calorimeter tests were also performed at a lower heat flux, namely 25 kW/m^2^. Due to the lower thermal power transmitted to the specimen, the time required to reach ignition temperature is extended [43], thereby enabling the protective action of the coating to be manifested.

**Figure 6:**
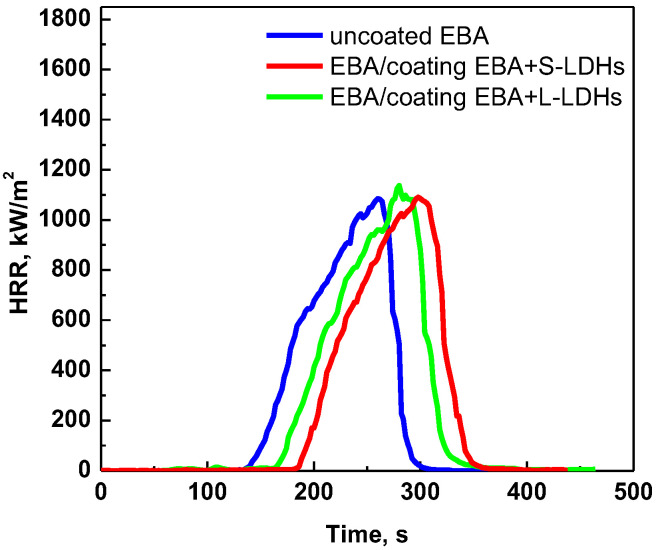
HRR as a function of time: cone calorimetry (heat flux = 25 kW/m^2^) curves of uncoated EBA and EBA-based surface-coated samples.

Figure 6 shows the HRR curves of EBA-based samples under a heat flux of 25 kW/m^2^, while Table 4 summarizes the main results and parameters (THR curves are reported in Figure S5). It can be clearly observed that, in this case, the surface-coated samples exhibit delayed TTI compared to the uncoated EBA. In this fire scenario (namely, at low heat flux), the thermo-oxidative degradation of EBA is slowed down; in fact, the TTI is 30% higher than at 30 kW/m^2^. Therefore, the LDH lamellae have the capacity to organize themselves in order to form a protective layer, thereby promoting the ignition delay.

Apart from the differences in TTI, for all investigated samples, the shape of the HRR curve remains almost unchanged compared to that of the uncoated copolymer. The THR of EVA and EBA/coating is higher with respect to the corresponding neat polymers due to the fact that the coated samples contain a higher amount of polymer. Additionally, it can be observed that, regardless of the copolymer matrix, the surface-coated samples show increased FPI and decreased FIGRA with respect to the corresponding uncoated substrates (see values reported in Table 3 and Table 4), basically due to the increase in TTI discussed earlier, indicating better performance in the evaluated scenarios.

## 4. Conclusions

In this work, a surface-coating approach for imparting flame retardancy to ethylene-based copolymers is proposed. Specifically, films based on EVA and EBA and containing two types of LDHs were firstly prepared through cast extrusion and then applied through compression molding to the corresponding matrix substrates. The preliminary characterization of the films highlighted the achievement of a homogeneous dispersion and distribution of the embedded nanofillers (which also appeared preferentially oriented along the machine direction) regardless of the matrix and notwithstanding the presence of some aggregates in EBA-based materials. Cone calorimeter tests at high heat flux (i.e., 30 kW/m^2^) demonstrated the effectiveness of the surface coating in delaying the time to ignition of EVA-based materials. For EBA-based coated samples, a less evident increase in TTI compared to the uncoated sample was observed. This result can be explained by considering two different aspects. Firstly, the lower polarity of EBA with respect to EVA resulted in poorer dispersion of the embedded nanofillers, making it more difficult to form the protective layer. On the other hand, the protective action of the coating was also hindered by the anticipated ignition of EBA, which hampered the organization of the dispersed LDH lamellae in a protective layer. To confirm this last assumption, for the EBA-based material, the cone calorimeter test was repeated at a lower heat flux (namely, 25 kW/m^2^). In this scenario, the slower thermo-degradation of the copolymer allowed the dispersed nanofillers to rearrange and form a more efficient protective shield, resulting in delayed ignition. Overall, the proposed coating strategy represents a promising and versatile approach to enhance the surface flame retardancy of EVA- and EBA-based systems. In particular, this methodology can be effectively exploited for the protection of cables and wires, which are commonly formulated using EVA or EBA as base polymers, providing improved fire performance without significantly altering the bulk properties or processability of the materials.

## Figures and Tables

**Table 1:** Main results of TGA analyses performed in air.

	T_onset_ [°C]	T_max_ [°C]	Residue @ 600 °C [%]
EVA	291.2	419.7	0
EVA + S-LDHs	310.3	428.9	1.44
EBA	281.5	367.0	0
EBA + S-LDHs	294.8	422.7	1.53

**Table 2:** Melting temperatures and crystallinity content for EVA- and EBA-based nanocomposite films.

	T_m1_ [°C]	T_m2_ [°C]	$\chi_{c}$ [%]
EVA	48	81	32
EVA + S-LDHs	48	85	31
EVA + L-LDHs	49	83	31
EBA	50	94	36
EBA + S-LDHs	51	94	34
EBA + L-LDHs	51	96	35

**Table 3:** Cone calorimetry (heat flux = 30 kW/m^2^) data and parameters of all investigated coated samples and pristine copolymers.

	TTI [s]	TTP [s]	FPI [m^2^s/kW]	FIGRA [kW/m^2^s]
EVA	101 ± 1	210 ± 2	0.0675 ± 0.0027	7.1 ± 0.3
EVA/coating EVA + S-LDHs	133 ± 3	252 ± 6	0.0910 ± 0.0015	5.8 ± 0.1
EVA/coating EVA + L-LDHs	135 ± 13	255 ± 5	0.0968 ± 0.0119	5.5 ± 0.2
EBA	91 ± 5	194 ± 12	0.0749 ± 0.0053	6.3 ± 0.5
EBA/coating EBA + S-LDHs	102 ± 2	208 ± 4	0.0836 ± 0.0036	5.9 ± 0.2
EBA/coating EBA + L-LDHs	107 ± 3	214 ± 2	0.0848 ± 0.0068	5.9 ± 0.3

**Table 4:** Cone calorimetry (heat flux = 25 kW/m^2^) data and parameters of uncoated and surface-coated EBA-based samples.

	TTI [s]	TTP [s]	FPI [m^2^s /kW]	FIGRA [kW/m^2^s]
EBA	124 ± 4	242 ± 9	0.1137 ± 0.0008	4.5 ± 0.4
EBA/coating EBA + S-LDHs	173 ± 9	307 ± 3	0.1470 ± 0.0200	3.9 ± 0.4
EBA/coating EBA + L-LDHs	161 ± 5	268 ± 9	0.1416 ± 0.0031	4.2 ± 0.1

## Data Availability

Dataset available on request from the authors.

## Supplementary Materials

The following supporting information can be downloaded at https://www.mdpi.com/article/10.3390/polym17233189/s1. Figure S1: DSC thermograms collected during the first heating scan for unfilled EVA and EVA-based nanocomposite films. Figure S2: DSC thermograms collected during the first heating scan for unfilled EBA and EBA-based nanocomposite films. Figure S3: THR curves for EVA and EVA-based specimens (heat flux = 30 kW/m^2^). Figure S4: THR curves for EBA and EBA-based specimens (heat flux = 30 kW/m^2^). Figure S5: THR curves for EBA and EBA-based specimens (heat flux = 25 kW/m^2^).
