# Supplementary material for: Layered Double Hydroxide Nanocomposite Coatings for Improved Flame Retardancy of Polyethylene-Based Copolymers
Source: Polymers (Basel). 2025 Nov 29;17(23):3189. doi: 10.3390/polym17233189 (PMC12694297; doi:10.3390/polym17233189)
Supplement: Supplementary file 1 [file polymers-17-03189-s001.zip › polymers-3987465-supplementary.pdf]

Supplementary material

Layered Double Hydroxide Nanocomposite Coatings for Improved Flame Retardancy of Polyethylene-based Copolymers

Giuseppe Trapani [1], Rossella Arrigo [1], Michele Sisani [2], Maria Bastianini [2] and Alberto Frache [1,*]

[1] Department of Applied Science and Technology, Politecnico di Torino, Viale Teresa Michel 5, 15121, Alessandria, Italy; giuseppe.trapani@polito.it, rossella.arrigo@polito.it, alberto.frache@polito.it

[2] Prolabin&Tefarm, Ponte Felicino (Perugia), Italy; michele.sisani@prolabintefarm.com, maria.bastianini@prolabintefarm.com

* Correspondence: alberto.frache@polito.it

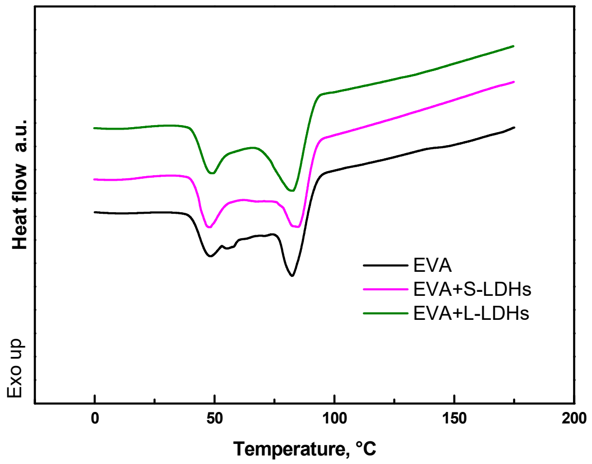

Figure S1. DSC thermograms collected during the first heating scan for unfilled EVA and EVA-based nanocomposite films

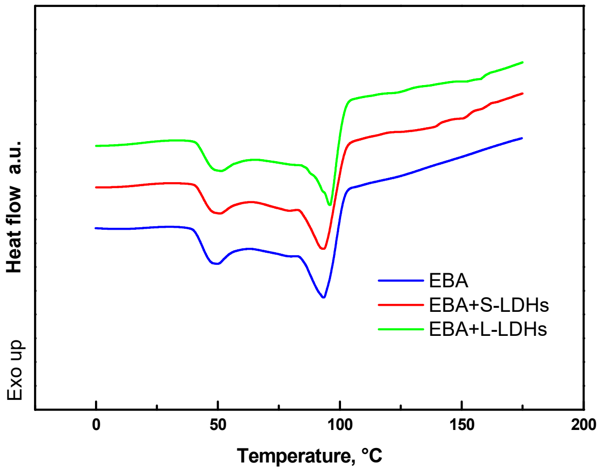

Figure S2. DSC thermograms collected during the first heating scan for unfilled EBA and EBA-based nanocomposite films

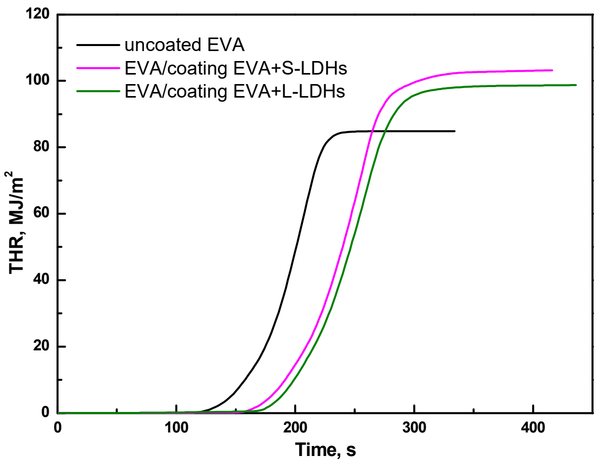

Figure S3. THR curves for EVA and EVA-based specimens (heat flux = 30 kW/m^2)

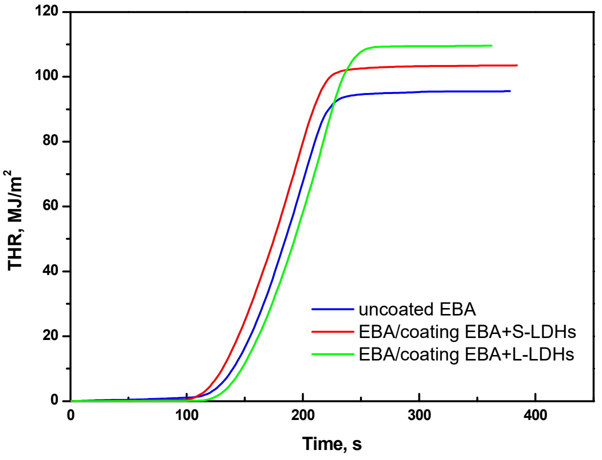

Figure S4. THR curves for EBA and EBA-based specimens (heat flux = 30 kW/m^2)

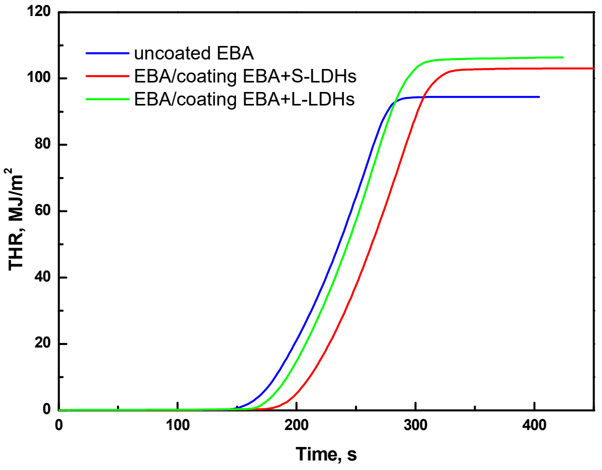

Figure S5. THR curves for EBA and EBA-based specimens (heat flux = 25 kW/m^2)
